# Effect of Supplementing Milk Replacer with *Boswellia serrata* Resin on Growth Performance, Serum Biochemical Profile, and Meat Quality of Suckling Lambs

**DOI:** 10.3390/ani16040626

**Published:** 2026-02-16

**Authors:** Bassam Abu Aziz, Halima Zoabi, Soha Ghzayal, Navid Ghavipanje, Ahmed Eid Kholif, Secundino Lopez, Hajer Ammar

**Affiliations:** 1 Veterinary Services and Animal Health Department, Jenin 009704, Palestine; bassamabuazez@yahoo.com (B.A.A.); st_halimeh@yahoo.com (H.Z.); 2 High Agriculture School of Kef, University of Jendouba, Jendouba 8100, Tunisia; soha2025.ghzayel@hotmail.com; 3 Laboratory of Agricultural Production System and Sustainable Development (SPADD), Higher Agriculture School of Mograne, University of Carthage, Zaghouan 1121, Tunisia; 4 Biotechnology Directorate, National Agricultural Research Center (NARC), Ministry of Agriculture, Jenin 009704, Palestine; 5 Department of Animal Science, Faculty of Agriculture, University of Birjand, Birjand 97175-331, Iran; panje@birjand.ac.ir; 6 South Khorasan Agricultural and Natural Resources Research and Education Center, Agricultural Research, Education and Extension Organization (AREEO), Birjand 97175-331, Iran; 7 Department of Animal Sciences, North Carolina Agricultural and Technical State University, Greensboro, NC 27411, USA; aekholif@ncat.edu; 8 Dairy Science Department, National Research Centre, 33 Bohouth St. Dokki, Giza 12622, Egypt; 9 Departamento Produccion Animal, Universidad de Leon, 24007 Leon, Spain; s.lopez@unileon.es; 10 Instituto de Ganadería de Montaña, CSIC-Universidad de León, Finca Marzanas, Grulleros, 24346 Leon, Spain

**Keywords:** *Boswellia serrata*, growth performance, biochemical profile, meat quality, phytogenic additives, sensory analysis

## Abstract

This study investigated the effects of supplementing a commercial milk replacer with *Boswellia serrata* resin (BSR), a natural frankincense extract rich in boswellic acids that possess anti-inflammatory and antioxidant properties, on the growth performance, serum biochemical parameters, and meat quality of suckling lambs. Twenty-four Assaf lambs were randomly assigned to three groups (*n* = 8 per group): natural suckling (NS), milk replacer (MR), and MR supplemented with BSR (MR+BSR). The experimental period lasted 60 days, during which growth performance and blood indices were evaluated, followed by meat quality assessment at the end of the trial. Lambs reared artificially (MR and MR+BSR) showed greater growth performance than naturally suckled lambs, as evidenced by significantly higher final body weight (*p* = 0.003) and average daily gain (*p* = 0.002), while initial body weight did not differ among treatments (*p* = 0.881). Within the artificially reared groups, however, BSR supplementation did not further increase growth relative to milk replacer alone. Supplementation with BSR improved the health and metabolic profile of lambs, resulting in higher serum total protein (*p* < 0.001), albumin (*p* < 0.001), and globulin (*p* < 0.001) concentrations. Moreover, BSR supplementation reduced the increase in aspartate aminotransferase (*p* < 0.001) and alanine aminotransferase (*p* < 0.001) activities observed in the MR group. No significant differences were detected in meat proximate composition (*p* > 0.05). However, meat from the NS group was significantly harder (*p* = 0.002) and more cohesive (*p* = 0.003) than meat from lambs fed MR or MR+BSR. In addition, the MR+BSR group produced meat with a higher redness (a*) value (*p* = 0.041), indicating an improved color quality. In conclusion, supplementing milk replacers with BSR enhanced the physiological and biochemical health of suckling lambs and enhanced specific meat quality traits, suggesting its potential as a natural functional additive in artificial rearing systems.

## Simple Summary

This study explored how adding a natural plant resin from *Boswellia serrata*, commonly known as frankincense, to milk replacers affects the growth, health, and meat quality of young lambs. Twenty-four lambs were divided into three groups: those that suckled naturally, those fed a commercial milk replacer, and those fed the same replacer with added *Boswellia serrata* resin. Over a 60-day period, researchers measured growth, blood health indicators, and meat characteristics. Lambs raised with milk replacer, with or without the resin supplement, grew faster and reached higher final weights than those suckled naturally, whereas the resin-supplemented group showed numerically, but not statistically, higher gains than lambs fed milk replacer alone. The supplemented group also showed better blood health, including improved protein levels and healthier liver function. While the basic composition and tenderness of the meat were similar among groups, meat from naturally suckled lambs was harder, whereas the resin-supplemented group produced meat with a more appealing red color. Overall, adding *Boswellia serrata* resin to milk replacers improved the wellbeing of lambs and enhanced some aspects of meat quality. This natural supplement could offer farmers a safe and sustainable way to raise healthy lambs when natural suckling is not possible.

## 1. Introduction

Newborn lambs experience multiple stressors during early life, including pathogen exposure, transportation, and weaning, which can suppress immune function, increase morbidity rates, and impair long-term productivity [1]. The syndesmochorial ovine placenta prevents prenatal transfer of immunoglobulins, making colostrum intake essential for passive immunity [2]. Accordingly, early nutrition plays a critical role in lamb survival, growth, immune maturation, and lifetime productivity [3,4].

While natural nursing provides an optimal balance of nutrients and immune-bioactive components (such as immunoglobulins and antimicrobial peptides) that support intestinal development and systemic resilience [5], many intensive production systems often rely on artificial milk replacers (MR) to manage large lambing cohorts. Although commercial MR provide adequate energy and protein, they typically lack the bioactive molecules present in maternal milk [6], which can compromise lamb immunity, gut function, and performance. This has intensified the search for natural feed additives capable of improving the functional quality of MR and supporting the “One Health” framework by reducing antimicrobial reliance in livestock [7,8]. Phytogenic feed additives have attracted particular interest due to their antioxidant, anti-inflammatory, and immunomodulatory properties [9,10]. Several studies have demonstrated their potential benefits in suckling lambs. A recent meta-analysis further confirmed that phytogenics (mostly from *Origanum vulgare*, *Salvia rosmarinus*, and *Allium sativum*) consistently enhance feed efficiency, health biomarkers, and meat quality in lambs [11], supporting their potential as functional supplements in neonatal diets.

Within this context, *Boswellia serrata* resin (BSR) has emerged as a promising phytogenic additive because of its rich content of boswellic acids and other triterpenoids with well-documented anti-inflammatory, antioxidant, antimicrobial, hepatoprotective, and immunomodulatory functions [12,13]. The resin contains terpenoids, phenolic compounds, flavonoids, and phenylpropanoids [13], including quercetin and kaempferol [14]. Among its various boswellic acids, 11-keto-β-boswellic acid (KBA) and 3-O-acetyl-11-keto-β-boswellic acid (AKBA) have been most widely studied due to their potent biological activities [12].

Evidence in monogastric species indicates that BSR supplementation enhances nutrient digestibility, hematological status, and immune function [15,16]. Although research in ruminants is limited, early-lactation goats supplemented with BSR showed improved metabolic profiles, lowered blood nonesterified fatty acid (NEFA) and β-hydroxybutyrate (BHBA) concentrations, reduced somatic cell count, without deleterious effects on intake and digestibility [17]. Moreover, Hashem et al. [18] showed that oral administration of BSR (2 or 4 g/day) for transition goats positively affected lipid metabolism, udder/uterine health, colostrum IgM concentration, and milk yield; effects ascribed to its capacity to modulate oxidative and inflammatory pathways. These effects are highly relevant to suckling lambs, as the transition from maternal milk to MR often induces intestinal stress, inflammation, and reduced nutrient utilization [5,6].

Despite the encouraging findings in other species, no studies have evaluated whether adding BSR to liquid MR can address the well-recognized challenges of artificial rearing, such as reduced immune support, greater susceptibility to intestinal inflammation, and the absence of bioactive compounds that influence metabolic stability and later meat quality, or whether it can enhance growth, systemic metabolism, or meat quality in artificially reared lambs. Therefore, this study was designed with a clearly defined hierarchical hypothesis framework. The main hypothesis (H1-main) was that supplementation of milk replacer with BSR would improve systemic metabolic and health-related indicators in artificially reared lambs compared with unsupplemented MR, as reflected by serum protein fractions and liver enzyme activities. Secondary hypotheses (H1-secondary) were that BSR supplementation would: (i) enhance growth performance parameters, and (ii) improve meat quality traits, including water-holding capacity, texture, and color stability. For each outcome, the corresponding null hypothesis (H0) stated that BSR supplementation would have no effect relative to control MR-fed lambs. These hypotheses were defined as a priori and guided the experimental design and statistical analyses.

## 2. Materials and Methods

This study was conducted on a private Assaf sheep farm in the Maithaloun village (32.4600° N latitude and 35.2900° E longitude, 200–300 m above sea level), located southeast of Jenin city within the Jenin governorate of Palestine. The region receives an average annual rainfall of approximately 400–550 mm, with temperatures ranging from 7 to 17 °C in winter to 28–33 °C in summer.

### 2.1. Ethical Approval, Animals, and Experimental Design

All experimental procedures were reviewed and approved by the Institutional Animal Care and Use Committee (IACUC) under project ID # 1834-22-2025 and conducted in compliance with ARRIVE guidelines for the ethical use of animals in research [19].

Twenty-four clinically healthy neonatal Assaf lambs [5.40 ± 0.62 kg body weight (BW, mean ± SD)] were assigned to three experimental groups (*n* = 8 per group) for an eight-week trial in a completely randomized design as follows: (1) natural suckling (NS), serving as the positive control, where lambs remained with their dams and had free access to maternal milk; (2) MR, serving as negative control, representing conventional artificial rearing practices, where lambs were isolated and fed exclusively on a commercial MR; and (3) MR supplemented with *Boswellia serrata* resin (MR+BSR), serving as the treatment group to investigate the potential of BSR to improve growth and health under artificial rearing conditions. This study did not analyze the chemical composition of BSR. The resin was administered as a whole powdered product rather than as a solvent-derived extract, ensuring that the complete spectrum of bioactive components was consistently delivered. However, prior research, such as Yang et al. [20], indicates that the resin mainly consists of alcohol-soluble components (60–80%), water-soluble gum (15–20%), and essential oils (5–7%). It contains bioactive compounds like α- and β-boswellic acids (10–21%) and their acetyl derivatives (0.05–6.0%). Additionally, certain active compounds, including 11-keto-β-boswellic acid (KBA; 2.5–7.5%) and 3-O-acetyl-11-keto-β-boswellic acid (AKBA; 0.1–3.0%), have been identified as contributors to the resin’s biological and pharmacological effects.

The three rearing systems differed by management conditions. NS lambs stayed with their dams in group pens. Lambs in the MR and MR+BSR groups were individually housed in sanitized pens (1.2 × 1.5 m) with rubber flooring, adequate ventilation, and shading to reduce environmental and social stress. All lambs received maternal colostrum within the first 3 days postpartum. From day 4 onward, NS lambs continued suckling, while MR and MR+BSR lambs were bottle-fed a commercial MR (26% CP, 18% fat; Agrofeed Ltd., Ramallah, Palestine) reconstituted at 150 g/L and offered twice daily (07:00 and 18:00).

For the MR+BSR group, the resin supplement was prepared using the whole powdered *Boswellia serrata* material. Each lamb received a precisely measured daily dose of BSR, following a stepwise supplementation protocol of 0.5 g/day during week 1, 1.0 g/day during weeks 2–3, and 2.0 g/day from week 4 until weaning (day 60). The required daily amount of resin was accurately weighed and hydrated in warm water for 12 h to facilitate dispersion, without any filtration to prevent loss of insoluble active fractions. The fully dispersed resin was then thoroughly incorporated into the morning milk replacer to ensure a uniform dose of the complete resin matrix. Because the resin was added based on its precise as-fed weight and all MR was consumed during bottle feeding, each lamb received the full intended amount of the active components without variation due to solubility differences. This preparation method was selected to reflect practical on-farm use. While the total intake of active compounds (e.g., boswellic acids) was not measured, the controlled preparation and administration procedure was designed to maintain consistency in the delivered dose. Future studies should quantify active compound concentrations and actual intake to eliminate potential confounding related to extraction method variability and resulting differences in bioactive concentration or bioavailability.

The concentrate feed was gradually introduced starting from the third week of age. All lambs were slowly transitioned to a commercial concentrate to promote rumen development [17,18]. The feed comprised crushed barley, soybean meal, and wheat bran, formulated to contain about 18% crude protein (CP), 3.5% ether extract (EE), and 9% crude fiber (CF) on a dry matter (DM) basis. Additionally, the diet was supplemented with a vitamin-mineral premix per kg: 10,000 IU of Vitamin A, 2000 IU of Vitamin D3, 30 mg of Vitamin E, and essential trace minerals. Table 1 summarizes the nutrient composition of the concentrate given to all experimental groups.

### 2.2. Growth Performance Metrics

Individual body weight (BW) was measured at birth (day 1) and at 10-day intervals (days 10, 20, 30, 40, 50, and 60) using a calibrated digital scale (TCS, HNOPMA, Xinxiang, Henan, China; accuracy ±0.01 kg). Total weight gain (TWG) was calculated as the difference between final (FBW) and initial BW (IBW), and average daily gain (ADG) derived as TWG divided by the number of days on trial. The feed conversion ratio (FCR) was calculated using the following equation: FCR = dry matter intake (DMI)/ADG. The FCR was estimated based on group-level milk replacer consumption reconstituted to a standardized concentration (150 g/L). Daily intake volumes were recorded and converted to dry matter equivalents using the manufacturer’s specifications. While individual intake variations within groups were not measured due to the practical constraints of ad libitum liquid feeding with suckling lambs, this approach provides valid comparative estimates of feed efficiency between treatment groups managed identically. FCR was calculated as estimated dry matter intake (DMI) divided by ADG.

### 2.3. Blood Sample and Serum Biochemical Analysis

Blood samples (5 mL per lamb) were collected via jugular venipuncture into serum separator tubes (BD Vacutainer^®^, Franklin Lakes, NJ, USA) after overnight fasting (prior to morning feeding) on days 30 and 60 to minimize diurnal and postprandial variation [21]. Serum was harvested following centrifugation at 3000× *g* (K1015 Micro Prime; Centurion Scientific Ltd., Stoughton, Chichester, UK) for 15 min at 4 °C, aliquoted into micro-tubes, and stored at −20 °C until analysis.

Serum metabolites were analyzed using an automated chemistry analyzer (BK-6310VET, BIOBASE^®^, Jinan, Shandong, China) and commercial diagnostic kits (SPINREACT, Girona, Spain). All assays were performed according to the manufacturer’s standardized colorimetric and enzymatic protocols. Prior to analysis, serum samples were allowed to thaw at 4 °C, gently mixed, and inspected for hemolysis. The analyzer was calibrated daily using the manufacturer-provided multi-level calibrators, and internal quality-control samples were run at the beginning and midpoint of each analytical batch to verify measurement accuracy. Serum samples were tested for liver function markers [Alanine aminotransferase (ALT, U/L), aspartate aminotransferase (AST, U/L), creatine kinase (CK), total bilirubin (TBIL, mg/dL), albumin (Alb, g/dL)]; kidney function indicators [urea (BUN, mg/dL), creatinine (Cr, µmol/L)]; energy and protein metabolism [glucose (Glu, mg/dL), total protein (TP, g/dL), globulin (GLOB, g/dL; calculated as TP minus Alb)]; and mineral balance [calcium (Ca, mmol/L)]. All samples were analyzed in triplicate, and the mean of the three readings was used for statistical analysis.

### 2.4. Meat Quality Assessment

At the end of the trial, all lambs were humanely slaughtered at a licensed abattoir in accordance with standard procedures. All carcasses were chilled and stored at 4 °C for 24 h, and samples of the *Longissimus dorsi* (LD) muscle (approximately 500 g) were taken from the left side of each carcass, between the 12th and 13th ribs.

Proximate chemical composition, including moisture, fat, protein, salt, and total collagen, was determined using Near-Infrared (NIR) Spectroscopy with a Food Scan™ Meat Analyzer (Foss Analytical, Hillerød, Denmark), calibrated against reference AOAC methods. It should be noted that the NIR-based collagen value reported herein represents total collagen content, as the instrument calibration is based on AOAC hydroxyproline-based quantification of total collagen. Texture Profile Analysis (TPA) was conducted on cylindrical cores (25 mm diameter, 15 mm height) excised parallel to the muscle fiber direction from LD muscle. Analyses were performed at room temperature (~22 °C) using a Brookfield Texture Analyzer (CT50, Middleboro, MA, USA) equipped with a 50 mm cylindrical aluminum probe, following a double-compression cycle adapted from de Huidobro et al. [22] for cooked meat. Samples (1.0 cm^3^, in triplicate) were subjected to double compression to 40% strain at a crosshead speed of 2.0 mm/s (pre-test, test, and post-test phases), trigger force 0.049 N with a 5 s dwell interval between compressions. TPA parameters were calculated as follows: Hardness = peak force (N) during the first compression, cohesiveness = [(area under second compression curve)/(area under first compression curve)], springiness = (distance recovered after the first compression)/(original sample height), gumminess = hardness × cohesiveness, and chewiness = gumminess × springiness [22]. Force-deformation curves were recorded at 0.01 s intervals via Texture Expert. Prior to analysis, all LD samples were trimmed of visible fat and connective tissue and equilibrated at room temperature (20–22 °C). Cylindrical cores (25 mm diameter × 15 mm height) were cut parallel to muscle fiber orientation to ensure uniformity. Each core was placed centrally on the platform of the Brookfield Texture Analyzer (CT50) (AMETEK Brookfield Engineering Laboratories, Middleboro, MA, USA) fitted with a 50 mm flat-ended aluminum probe. The probe performed a two-cycle compression test to 40% of the original sample height at a constant crosshead speed of 2.0 mm/s for the pre-test, test, and post-test phases, with a 5 s interval between compressions to allow partial structural recovery. Force–time curves were recorded for each cycle, and the instrument software automatically integrated the curve areas, peak forces, and displacement values to derive TPA parameters.

For pH analysis, muscle samples (3 g, in triplicate) were homogenized with 20 mL deionized water using a blender (Model 8011ES, Waring^®^, Torrington, CT, USA), and pH (24 h post-mortem) was measured in triplicate with a pH meter (Model HI98127, Hanna^®^, Woonsocket, RI, USA) [21].

Meat color was assessed using the CIELAB color space [23] with a HunterLab colorimeter (Model D25 with DP-9000 optical sensor, HunterLab, Reston, VA, USA) on the cut surface of 2.5 cm-thick, fat-free muscle samples. Color coordinates including lightness (L*), redness (a*), and yellowness (b*) were measured in triplicate. Drip loss was calculated by suspending ~30 g meat samples in sealed containers at 4 °C for 24 h, with loss expressed as % weight reduction. Cooking loss was determined by cooking 30 g samples in vacuum-sealed bags at 71 °C for 30 min. Results were reported as % weight loss relative to raw weight [24].

### 2.5. Statistical Analysis

Data were analyzed using IBM SPSS (v.28, Armonk, NY, USA). Normality and homogeneity of variances were confirmed using the Shapiro–Wilk and Levene’s tests, respectively. A one-way analysis of variance (ANOVA) was performed to evaluate the effect of dietary treatment (NS, MR, MR+BSR) on all response variables, with treatment as the fixed factor. The IBW was a co-variable to adjust the DMI, FBW, TWG, and ADG. When significant differences were observed (*p* < 0.05), post hoc comparisons among group means were conducted using Duncan’s Multiple Range Test. Results were expressed as least squares mean (LSM) ± standard error of the mean (SEM). Significant differences were considered when *p* < 0.05, and a trend was considered when *p* ≥ 0.05 but ≤0.10.

## 3. Results

### 3.1. Growth Performance

**Figure 1:**
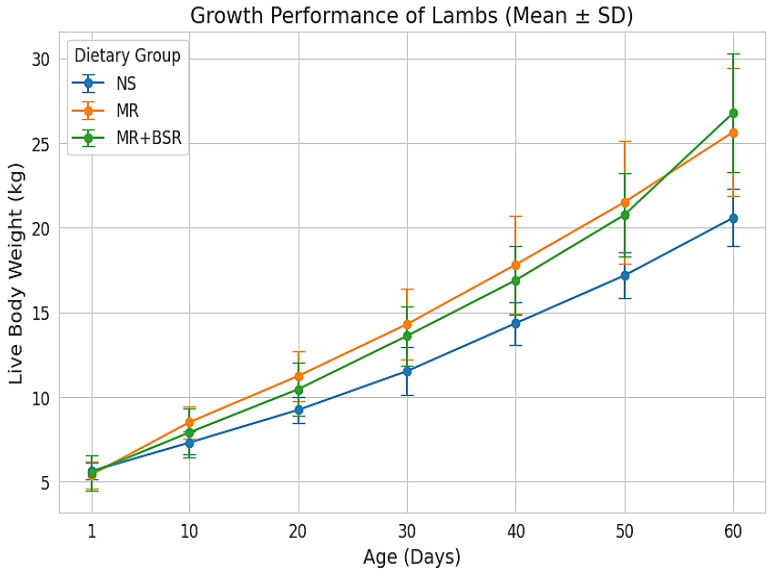
Mean live body weight (kg) of Assaf lambs from birth to weaning (60 days) under natural suckling (NS), milk replacer (MR), and BSR supplementation (MR+BSR). Error bars represent the standard error of the mean (SEM). Significance levels: Treatment: *p* < 0.001, Time: *p* < 0.001, Treatment × Time: *p* < 0.001.

Over the 60-day trial, dietary treatments exerted significant effects on lamb growth performance (Table 2 and Figure 1). The IBW (*p* = 0.881) did not differ significantly among the treatment groups. However, the FBW (*p* = 0.003), TWG (*p* = 0.002), and ADG (*p* = 0.002) were higher in lambs reared artificially (MR and MR+BSR groups) compared to the NS group. Although the inclusion of BSR numerically improved growth parameters, the differences between the MR and MR+BSR groups were not statistically significant (*p* > 0.05), suggesting that BSR supplementation supported comparable growth efficiency to standard MR feeding.

### 3.2. Serum Biochemical Parameters

Serum concentrations of total protein (*p* < 0.001), albumin (*p* < 0.001), globulin (*p* < 0.001), glucose (*p* = 0.039), and calcium (*p* < 0.001) were significantly higher in the MR+BSR group compared to both the MR and NS groups (Table 3). In contrast, blood urea nitrogen concentration was significantly lower in the MR+BSR group (*p* = 0.021). The activities of liver enzymes AST (*p* < 0.001) and ALT (*p* < 0.001) were higher in the MR and NS groups compared to the MR+BSR group, indicating that BSR supplementation may have exerted a hepatoprotective effect. The enzyme creatine kinase (*p* = 0.001) was higher in the NS group compared to the MR and MR+BSR groups. However, serum creatinine concentration remained unaffected by dietary treatments (*p* = 0.158).

### 3.3. Meat Quality Characteristics

**Figure 2:**
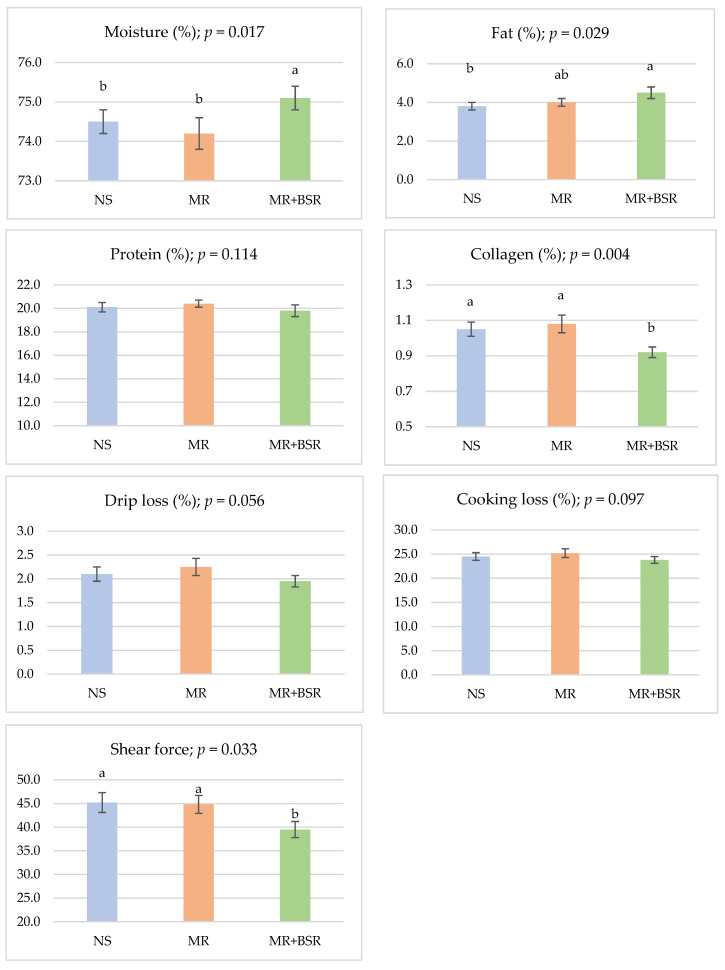
Mean values of physicochemical properties of *Longissimus dorsi* muscle in Assaf lambs under natural suckling (NS), milk replacer (MR), and BSR supplementation (MR+BSR). ^a,b^ Means for each parameter with different superscript letters differ significantly (*p* < 0.05).

The effects of dietary treatments on the proximate composition and physical quality parameters of meat are presented in Figure 2. Moisture content differed significantly (*p* = 0.017), with the MR+BSR group showing a slight but consistent increase compared with the NS and MR groups. Fat content also varied among treatments (*p* = 0.029), being higher in the MR+BSR group, whereas protein content remained statistically unaffected (*p* = 0.114).

**Figure 3:**
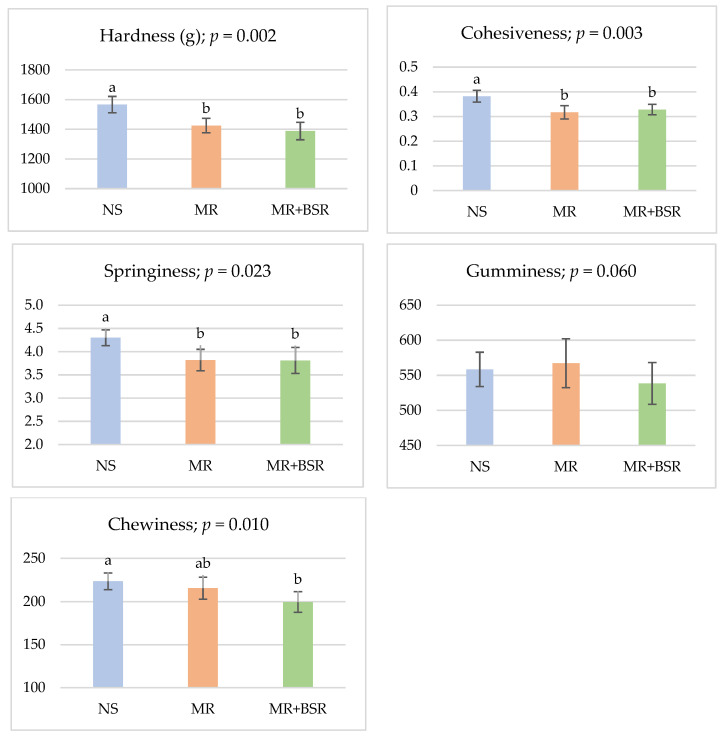
Mean values of Texture Profile Analysis (TPA) parameters of *Longissimus dorsi* muscle in Assaf lambs under natural suckling (NS), milk replacer (MR), and BSR supplementation (MR+BSR). ^a,b^ Means for each parameter with different superscript letters differ significantly (*p* < 0.05).

Total collagen percentage was significantly reduced (*p* = 0.004) in the MR+BSR group relative to the other groups. Although drip loss (*p* = 0.056) and cooking loss (*p* = 0.097) were not significantly affected, both parameters tended to decrease in meat from MR+BSR-fed lambs. Furthermore, shear force was significantly lower (*p* = 0.033) in the MR+BSR group, indicating improved tenderness associated with BSR supplementation. The Texture Profile Analysis (TPA) of the *Longissimus dorsi* muscle is presented in Figure 3. Muscle from the NS group was significantly harder (*p* = 0.002), more cohesive (*p* = 0.003), and springier (*p* = 0.023) compared to muscle from lambs reared on MR (MR and MR+BSR groups). No significant differences were observed between the MR and MR+BSR groups for these parameters, and gumminess was similarly unaffected (*p* = 0.060). These results suggest that factors other than total collagen, particularly those related to post-mortem muscle biochemistry, likely contributed to the reduced shear force observed in the MR+BSR group.

Instrumental color analysis of the *Longissimus dorsi* muscle indicated that dietary treatments had no significant effects on lightness (L*; *p* = 0.819) or yellowness (b*; *p* = 0.472) (Table 4). However, noticeable differences were observed in ultimate pH (24 h post-mortem), where the MR group showed a higher value (*p* = 0.032) than both the NS and MR+BSR groups. The elevated pH in MR-fed lambs may reflect a greater reliance on glycogen reserves at slaughter or altered energy metabolism linked to artificial rearing, which can limit post-mortem acidification. In contrast, the MR+BSR group maintained a pH comparable to naturally suckled lambs, suggesting that BSR supplementation may have helped stabilize muscle energy status and prevent excessive pH elevation. Redness (a*; *p* = 0.041) was higher in the meat from lambs supplemented with BSR (MR+BSR group) compared to both the NS and MR groups, which is consistent with the more favorable pH range that supports oxygenation and color development.

## 4. Discussion

Early-life nutrition is critical for supporting immune development, metabolic health, and long-term productivity in lambs [1,5]. In response to consumer demand for natural, antibiotic-free, and eco-friendly animal products, phytogenic compounds have emerged as promising natural alternatives to enhance both animal health and meat quality [7,11,21]. The present study evaluated the impacts of supplementing a commercial MR with BSR from birth to weaning in lambs, comparing outcomes against NS or unsupplemented MR. Consistent with the growth data, lambs reared on a milk replacer (MR and MR+BSR) exhibited significantly higher final body weight, total weight gain, and average daily gain than naturally suckled lambs, confirming that artificial rearing per se was the primary driver of enhanced growth performance. However, within the artificially reared groups, supplementation with BSR did not confer additional growth advantages beyond those achieved with milk replacer alone. Despite the lack of a growth-promoting effect, BSR supplementation yielded notable improvements in hematological indicators and meat quality traits, particularly reduced shear force and improved color stability. These findings indicate that while milk replacer underpins pre-weaning growth enhancement, BSR specifically contributes to improved physiological resilience and meat quality without further stimulating growth. Importantly, although artificial rearing may not fully replicate the benefits of maternal nursing, the strategic inclusion of phytogenic compounds such as BSR may partially alleviate its physiological limitations and enhance product quality without compromising growth. 

### 4.1. Growth Performance

The preweaning period represents a key window for programming long-term productivity in meat lamb systems [1,25]. In the present study, growth indicators including FBW, TWG, and ADG were significantly higher in artificially reared lambs (MR and MR+BSR) compared with naturally suckled lambs, confirming that controlled feeding with MR can provide a more consistent nutrient supply during early development. However, the absence of additional gains in the MR+BSR group relative to the MR group indicates that the tested BSR level (0.3 g/L) did not further stimulate growth efficiency beyond the effect of milk replacer itself under the current conditions. This observation is consistent with earlier work in Assaf lambs, which reported comparable weaning weights (at 60 days of age) between NS and MR groups [26].

Supplementation of BSR in lambs did not negatively affect growth performance relative to milk replacer alone, indicating that it is safe for neonatal ruminants and does not impair nutrient assimilation. Given that ewe milk is the exclusive dietary source for neonatal lambs, its quantity and quality directly shape preweaning outcomes [3]. While energy intake is the principal driver of early growth (a factor not measured here) suboptimal milk provision would be expected to impair both weight gain and viability during this sensitive phase [1].

The effects of BSR on energy partitioning and utilization in sheep remain poorly characterized. In the present study, BSR was supplemented in a stepwise manner—0.5 g/day in week 1, 1.0 g/day in weeks 2–3, and 2.0 g/day from week 4 until weaning—to ensure gradual adaptation and to avoid metabolic disturbances. While direct measurements of metabolizable energy were not conducted, the absence of negative effects on feed intake, ADG, or FBW suggests that this supplementation regimen did not impair energy utilization. Studies with other plant-derived additives or extracts reported that metabolizable energy intake in ewes either remain unaffected [25] or increases [27] with *Moringa oleifera* leaf extract supplementation, though, efficacy depends strongly on the phytochemical composition and administration regimen. Confirming our results, Cordero-Mora et al. [28] demonstrated that graded levels of a polyherbal additive (0, 0.25, 0.50, and 1.0 g/day) administered over a 60-day nursing period to Hampshire × Suffolk crossbred lambs did not negatively influence growth metrics (i.e., FBW and ADG). Similarly, oral administration of suckling lambs with red orange and lemon extract (90 mg/kg of live weight) from birth until slaughter did not impair growth performance or FBW [29]. These findings support the notion that carefully dosed bioactive plant additives, including BSR, are unlikely to disrupt energy metabolism in young lambs. 

Additionally, supplementation of lambs with pepper extract (at 300 mg/kg) had no effect on weight gain, ADG, or DMI [30]. It has been suggested that low doses of secondary metabolites in the polyherbal blend may enhance productivity, whereas higher doses can suppress growth, likely due to the antinutritional or toxic effects of compounds such as saponins, alkaloids, terpenes, and tannins [28]. Furthermore, feed palatability and intake are highly sensitive to the phytogenic source, its concentration, plant-specific phytochemistry, and interactions with diet composition, administration timing, production stage, and individual lamb traits [28]. The absence of any additional growth response to BSR beyond that achieved with MR in the present study contradicts the theoretical expectation that dietary phytochemicals, at moderate concentrations, facilitate more efficient nitrogen utilization and thereby enhance productivity [11]. The lack of growth stimulation in the present study is consistent with the hypothesis that low-to-moderate doses of plant secondary metabolites, such as boswellic acids, exert subclinical protective effects (e.g., hepatoprotection, reduced inflammation) without altering anabolic drive. This is further supported by our serum data, which show significantly improved protein status and reduced liver enzyme activity in the MR+BSR group, despite unchanged ADG. Thus, BSR appears to enhance physiological resilience during artificial rearing, even in the absence of growth promotion. However, Giller et al. [31] highlighted that polyphenolic compounds may exert different effects during the suckling phase compared with post-weaning periods. Furthermore, the marked heterogeneity in methodological approaches, including basal diet composition, polyphenol class, dosage, duration, and onset of administration, likely explains the inconsistent growth responses reported in the literature [7,31]. The current study did not measure energy balance, milk replacer intake, nutrient digestibility, or metabolizable energy consumption in the lambs, which limits our ability to conclusively determine whether the superior growth performance observed in both artificially reared groups (MR and MR+BSR) relative to NS lambs is attributable primarily to greater energy intake under milk-replacer feeding or to other management-related factors; importantly, the lack of differences between MR and MR+BSR indicates that this advantage cannot be ascribed to BSR supplementation itself. Future research should incorporate comprehensive assessments of feed intake, energy balance, and nutrient utilization to fully elucidate the mechanistic basis of BSR’s influence on early-life nutrition and growth efficiency.

### 4.2. Serum Biochemical Parameters

Serum biochemical profiles are commonly employed to evaluate the systemic impact of phytogenic compounds on metabolic and immune homeostasis [28]. In the present trial, serum biochemical parameters support the hypothesis that BSR improves the overall health status of lambs.

Lambs supplemented with BSR had a 7.56% higher plasma glucose level compared to those in the NS group. Mahdian et al. [32] reported that the main characteristic of the mechanism of action of BSR involves controlling metabolic syndrome and related disorders by decreasing insulin resistance, consequently enhancing plasma glucose circulation and regulating peripheral glucose uptake.

Serum total protein and its globulin fraction are clinically relevant metrics for evaluating humoral immunity and systemic health in neonatal lambs [33]. Given that globulins constitute a major pool of circulating immunoglobulins and immune mediators, an increase in their concentration is frequently indicative of an activated or enhanced immune system [33]. It has been well established that lambs allowed to suckle naturally from the ewe show higher serum TP and GLOB in the first weeks than those artificially fed MR [34]. The results concerning serum proteins were particularly noteworthy; TP, albumin, and GLOB were increased by 43.3%, 29.8%, and 71.9%, respectively, in the BSR-supplemented group compared to the NS group, indicating enhanced hepatic synthetic capacity and potentially a strengthened immune response via globulins. This suggests that BSR not only protects against stress but may also promote positive metabolic processes. This response is consistent with previous research showing that *Boswellia serrata* resin stimulates hepatic metabolic activity and improves immune function through its anti-inflammatory and antioxidant bioactive compounds, particularly boswellic acids [17,18]. Such compounds reduce oxidative stress and inflammatory signaling, thereby improving liver efficiency and supporting the synthesis of circulating proteins. Furthermore, the reduction in AST and ALT in the MR+BSR group indicates better hepatocellular integrity, which may also contribute to the higher circulating protein fractions. Together, these mechanisms provide a plausible explanation for the >43% rise in total protein observed in BSR-supplemented lambs. Similarly, Hashem et al. [18] reported that dietary BSR ingestion (0, 2, or 4 g/day) from week 2 prepartum to week 7 postpartum in transition dairy goats significantly improved blood plasma albumin compared with control throughout the experimental period, except at week 3 postpartum. Moreover, dietary BSR supplementation (0, 200, 400, or 600 mg/kg diet) stimulated hepatic protein synthesis, as evidenced by 55%, 53%, and 54% increases in serum albumin, globulins, and total proteins, respectively, along with a 154% increase in interleukin-10 of broilers [15], which the authors attribute to improved liver synthetic function. In the present study, the concurrent elevation of both albumin and globulin, together with decreased AST and ALT values in MR+BSR lambs, supports the interpretation that the increase in total protein reflects improved hepatic integrity and heightened immunoglobulin synthesis.

The significant elevation of liver enzymes such as AST and ALT in the MR group is a classic marker of metabolic stress associated with artificial feeding [34]. The marked reduction in liver-related enzymes in the BSR-supplemented lambs to levels comparable to the NS group suggests a hepatoprotective effect. This aligns with the documented anti-inflammatory, antioxidative, and hepatoprotective properties of BSR [14,35].

It is well established that the hepatoprotective impacts of BSR are primarily linked to pentacyclic triterpenoids collectively called boswellic acids, which protect against multiple hepatotoxins through overlapping cellular mechanisms [14]. Specifically, BSR bioactive compounds upregulate Nrf2/HO-1 and downregulate NF-κB, CYP2E1, and NOX1/2/4 signaling pathways collectively reduced inflammation and supporting liver function [36]. In addition, limonene, the major monoterpene component of BSR, suppresses matrix metalloproteinase and its mRNA, exerting anti-inflammatory and antioxidant effects relevant to ulcerative colitis [37]. Bisabolene, the predominant sesquiterpene identified in BSR, was found to modulate inflammatory responses and exert selective cytotoxic effects against mammary tumor cells, leading to its investigation in breast cancer treatment [38]. Additionally, α- and β-amyrins were reported to ameliorate cerulein-induced acute pancreatitis via their antioxidant and anti-inflammatory actions [39].

Moreover, BSR protects Balb/cA mice from acetaminophen (APAP)-induced liver damage, by suppressing the expression and catalytic activity of cytochrome P450 2E1, thereby reducing the generation of the toxic APAP metabolite NAPQI. The anti-inflammatory efficacy of BSR is further evidenced by its inhibition of NF-κB p65 nuclear translocation and JNK phosphorylation, leading to diminished secretion of pro-inflammatory cytokines and chemokines [35]. Our findings extend this hepatoprotective role to neonatal lambs, suggesting that BSR helps safeguard liver integrity during the metabolic challenges of artificial rearing.

### 4.3. Meat Quality Characteristics

Meat quality holds significant practical relevance for the meat industry, as it directly influences consumer preferences and helps minimize economic losses associated with weight loss during processing and storage [40]. Sheep meat composition varies according to animal age, slaughter weight, fat content, and feed type [29].

Our results showed no differences in LD muscle composition (i.e., DM, CP, EE, and ash content); however, BSR tended to decrease drip loss. The current literature lacks data on the effects of BSR on meat quality traits in lambs or other ruminant species. However, our results are in agreement with Dorantes-Iturbide et al. [21], who demonstrated that phytogenic formulations do not alter the fundamental nutritional composition of ovine meat. Similarly, no differences were observed in the proximate composition of the *Longissimus dorsi* muscle following dietary administration of the polyherbal additive at graded levels (0, 1, 2, and 3 g/kg) [41].

Regarding BSR application in monogastric species, Al-Yasiry et al. [42] showed that BSR supplementation (1.5, 2, and 2.5%) in broilers did not alter DM, CP, and ash content in breast and drumstick muscle. Our findings are also supported by Kiczorowska et al. [43], who reported increased WHC and reduced cooking losses in BSR-treated broilers.

Lower shear force values in MR+BSR-supplemented group are highly desirable from both consumer and industry perspectives. This improvement can be attributed to the preservation of muscle cell membrane integrity, which is often compromised by oxidative stress during the post-mortem conversion of muscle to meat. The antioxidant capacity of BSR is not limited to boswellic acids but also includes other triterpenoid compounds, flavonoids, and phenolic constituents that collectively scavenge reactive oxygen species and stabilize cellular membranes [14,40]. These bioactive compounds reduce fluid leakage, limit protein oxidation, and maintain myofibrillar structure, thereby contributing to lower shear force. Additionally, improvements in tenderness may occur through mechanisms independent of collagen content, including enhanced post-mortem proteolysis, reduced oxidative inhibition of the calpain system, and better preservation of myofibrillar proteins [44,45]. These pathways can reduce shear force even when total collagen levels remain unchanged. Similarly, the improvement in tenderness may result from enhanced post-mortem proteolytic activity or connective tissue cross-linking [46].

The MR group exhibited a significantly higher ultimate pH compared with both the NS and MR+BSR groups, although all values remained within the normal range for lamb LD muscle (5.5–5.8). Elevated pH, which reflects reduced post-mortem acidification, often results from depleted muscle glycogen or inhibited glycolysis and is associated with changes in meat color and WHC [47]. Earlier work has linked such patterns to metabolic stress or feeding-related shifts in energy reserves. The higher pH observed in the MR group may therefore reflect differences in muscle glycogen status or stress responses linked to feeding management or the metabolic effects of the MR regimen [48]. Lambs reared artificially often face greater physiological stress, which can reduce glycogen stores and slow lactic acid formation after slaughter. In addition, milk replacer–based diets can modify rumen development and systemic energy metabolism, potentially influencing glycogen deposition in skeletal muscle. Meanwhile, the MR+BSR group showed pH values similar to NS, suggesting that BSR supplementation helped counteract this metabolic disruption—possibly through improved antioxidant balance and reduced stress, supporting a more typical rate of post-mortem glycolysis. This aligns with previous findings that phytogenic antioxidants can help maintain muscle energy reserves and stabilize pH decline after slaughter.

While direct mechanistic evidence in lambs is limited, *Boswellia serrata* and its boswellic acids are known to exert anti-inflammatory and metabolic effects in livestock and have been linked to improvements in meat quality [16]. It has been well documented [49] that dietary supplementation with a phytogenic blend rich in triterpenoids and polyphenols reduced pre-slaughter plasma cortisol and muscle oxidative stress markers in lambs, thereby preserving muscle glycogen stores and promoting a more complete post-mortem pH decline. Given that boswellic acid, the primary bioactive constituent of BSR, modulate key inflammatory pathways (e.g., NF-κB and JNK) and upregulate endogenous antioxidant systems (e.g., Nrf2/HO-1) [14,35], it is plausible that BSR helps preserve muscle glycogen and support normal post-mortem glycolysis. Nevertheless, further studies are needed to explore muscle glycogen, stress, and inflammation biomarkers.

Meat color directly shapes consumer perception about quality and safety. Any noticeable discoloration is often interpreted as a sign of spoilage and results in reduced purchase intent and economic devaluation of the product [40,46]. In this study, LD muscle color was partially improved in BSR-supplemented lambs compared to both NS and MR groups, as evidenced by comparable lightness (L*) and yellowness (b*) values but significantly higher redness (a*). This shift toward greater red intensity may reflect improved oxidative stability of myoglobin pigments, a trait closely associated with perceived freshness and marketability [40].

The a* values in fresh meat depend on the myoglobin pigment being in its reduced oxymyoglobin state. Oxidative stress leads to oxidation of this pigment into the undesirable brown-colored metmyoglobin [50]. Plant-based additives have repeatedly demonstrated efficacy in improving meat oxidative stability by enhancing systemic antioxidant capacity and suppressing lipid peroxidation, these compounds effectively lower the accumulation of secondary oxidation products [29]. It has been well established that natural antioxidants from plants such as *D. ambrosioides* (L.) *C. longa* L., *P. nigrum* L., *N. velutina* Wooton, *Q. alba* L., *S. rosmarinus* Spenn., *S. aromaticum* L., *T. vulgaris* L., and *T. foenum-graecum* L. have shown efficacy in delaying discoloration through myoglobin stabilization [46]. Although there is no comparable data regarding BSR application in ruminants, better instrumental color in broiler meat following BSR supplementation is confirmed by Kiczorowska et al. [43]. Our results thus position BSR as a novel, natural strategy to enhance the visual appeal and oxidative stability of lamb meat, directly addressing a key industry challenge.

The physiological response to phytogenic additives like BSR differs markedly between ruminants and monogastric animals because of their contrasting digestive systems. In monogastrics, bioactive compounds are absorbed directly in the small intestine, while in ruminants they undergo extensive microbial fermentation and transformation in the rumen before any absorption occurs. This helps clarify why some responses seen in poultry—such as notable growth promotion [15]—did not appear in our lambs. In ruminants, the main advantages of BSR seem to be metabolic and health-related, including hepatoprotective and immunomodulatory effects reported in goats [17,18] and reflected in our findings, rather than direct improvements in growth. These differences highlight the need for species-specific research and caution against extrapolating results from monogastric systems to ruminant nutrition.

From an economic perspective, the use of BSR supplementation in an artificial-rearing system should be evaluated in terms of cost per lamb relative to the productivity gains. In our study, the BSR dose increased stepwise to reach 2.0 g/day per lamb. To illustrate feasibility, assuming the price of crude Boswellia resin is approximately US $20–30 per kilogram (i.e., ~US $0.02–0.03 per gram, depending on quality and supplier), the cost of BSR supplementation would be about US $0.04–0.06 per lamb per day at the 2.0 g/day level. Over an 8-week rearing period, this corresponds to roughly US $2.5–4.0 extra cost per lamb. If the observed improvements in meat quality, health status, or growth performance translate into higher market value (e.g., better price for more tender meat or reduced culling), this modest addition could be economically justified. However, cost–benefit feasibility will depend heavily on local resin market price, scale of production, lamb value, and whether the benefits persist post-weaning. Future studies should include a formal economic analysis comparing added feed costs to the economic gains from improved performance and meat quality, to guide adoption by producers.

## 5. Conclusions

This study evaluated the effects of artificial rearing with milk replacer, with or without *Boswellia serrata* resin (BSR) supplementation, on growth performance, metabolic status, and meat quality of suckling lambs compared with natural suckling. Artificially reared lambs (MR and MR+BSR) exhibited significantly higher final body weight, total weight gain, and average daily gain than naturally suckled lambs, demonstrating that milk replacer feeding enhanced preweaning growth performance. However, BSR supplementation did not provide additional growth benefits beyond those achieved with milk replacer alone; therefore, the null hypothesis (H0) related to BSR-induced growth enhancement was accepted. In contrast, BSR supplementation markedly improved systemic metabolic and health-related indicators, as evidenced by increased serum total protein, albumin, and globulin concentrations and reduced activities of liver enzymes (AST and ALT). Accordingly, the main alternative hypothesis (H1-main) was accepted. Furthermore, BSR supplementation improved selected meat quality attributes, including reduced shear force, lower drip loss, and enhanced redness (a*), without altering proximate composition, supporting acceptance of the secondary alternative hypothesis (H1-secondary) related to meat quality. Overall, these findings demonstrate that while artificial rearing with milk replacer is the primary driver of enhanced growth performance, BSR supplementation confers additional metabolic and meat quality benefits without compromising growth. BSR therefore represents a promising natural functional additive for improving physiological resilience and product quality in artificially reared lambs.

## Figures and Tables

**Table 1:** Chemical composition of concentrate fed to lambs (as provided by the feed manufacturer).

Chemical Analysis	Percentage (%)
Total Protein	18.0
Rumen Degradable Protein	11.1
Ash	6.6
RUP (Rumen Undegradable Protein)	6.2
Fiber	5.6
Fat	3.0
Calcium	0.95
Phosphorus	0.487

**Table 2:** Effect of natural suckling (NS), milk replacer (MR), and BSR supplementation (MR+BSR) on growth performance in Assaf lambs ^1^.

Parameter ^2^	Treatments ^3^			*p*-Value
	NS	MR	MR+BSR	
IBW, kg	5.62 ± 0.49	5.40 ± 0.79	5.53 ± 1.05	0.881
Day 10	7.30 ± 0.67	8.50 ± 0.97	7.90 ± 1.45	0.312
Day 20	9.23 ± 0.76 ^b^	11.23 ± 1.47 ^a^	10.45 ± 1.56 ^ab^	0.043
Day 30	11.52 ± 1.42 ^b^	14.29 ± 2.11 ^a^	13.60 ± 1.76 ^a^	0.031
Day 40	14.35 ± 1.27 ^b^	17.81 ± 2.87 ^a^	16.89 ± 2.04 ^a^	0.047
FBW, kg	20.58 ± 1.69 ^b^	25.64 ± 3.76 ^a^	26.78 ± 3.52 ^a^	0.003
TWG, kg	14.96 ± 1.55 ^b^	20.24 ± 3.11 ^a^	21.25 ± 3.20 ^a^	0.002
ADG, g/d	249.3 ± 25.8 ^b^	337.3 ± 51.8 ^a^	354.2 ± 53.3 ^a^	0.002

^1^ Values are the least squares means (LSM) ± standard error of the mean (SEM). ^a,b^ Means within a row with different superscript letters differ significantly (*p* < 0.05). ^2^ IBW, initial body weight; FBW, final body weight; TWG, total weight gain; ADG, average daily gain. ^3^ NS, natural suckling (positive control); MR, milk replacer (negative control); MR+BSR, milk replacer supplemented with *Boswellia serrata* resin (BSR).

**Table 3:** Effect of natural suckling (NS), milk replacer (MR), and BSR supplementation (MR+BSR) on some blood parameters in Assaf lambs ^1^.

Parameter ^2^	Treatments ^3^			*p*-Value
	NS	MR	MR+BSR	
TP, g/dL	50.26 ± 1.22 ^b^	50.17 ± 1.15 ^b^	72.00 ± 1.85 ^a^	<0.001
ALB, g/dL	26.91 ± 0.88 ^b^	28.01 ± 0.95 ^b^	35.25 ± 1.05 ^a^	<0.001
GLOB, g/dL	23.56 ± 0.75 ^b^	22.91 ± 0.68 ^b^	40.50 ± 1.12 ^a^	<0.001
AST, U/L	89.37 ± 2.45 ^b^	89.87 ± 2.91 ^a^	87.50 ± 2.66 ^b^	<0.001
ALT, U/L	27.12 ± 0.98 ^a^	27.25 ± 1.03 ^a^	25.62 ± 0.85 ^b^	<0.001
BUN, mg/dL	2.66 ± 0.11 ^b^	2.68 ± 0.14 ^b^	2.56 ± 0.09 ^a^	0.021
Cr, µmol/L	41.55 ± 1.35	42.23 ± 1.48	40.60 ± 1.29	0.158
Glu, mg/dL	4.10 ± 0.15 ^b^	4.18 ± 0.17 ^b^	4.41 ± 0.19 ^a^	0.039
CK, U/L	80.00 ± 2.51 ^a^	78.87 ± 2.84 ^b^	77.50 ± 2.11 ^b^	0.001
Ca, mmol/L	2.58 ± 0.08 ^b^	2.56 ± 0.07 ^b^	2.79 ± 0.09 ^a^	<0.001

^1^ Values are least squares means (LSM) ± standard error of the mean (SEM). ^a,b^ Means within a row with different superscript letters differ significantly (*p* < 0.05). ^2^ TP, total protein; ALB, albumin; GLOB, globulin; AST, aspartate aminotransferase; ALT, alanine aminotransferase; BUN, blood urea nitrogen; Cr, creatinine; Glu, glucose; CK, creatine kinase; Ca, calcium. ^3^ NS, natural suckling (positive control); MR, milk replacer (negative control); MR+BSR, milk replacer supplemented with *Boswellia serrata* resin (BSR).

**Table 4:** Effect of natural suckling (NS), milk replacer (MR), and BSR supplementation (MR+BSR) on color values of *Longissimus dorsi* muscle in lambs ^1^.

Parameter	Treatments ^2^			*p*-Value
	NS	MR	MR+BSR	
pH	5.65 ± 0.09 ^b^	5.85 ± 0.12 ^a^	5.70 ± 0.10 ^b^	0.032
Lightness (L*)	54.86 ± 0.55	54.97 ± 0.61	55.14 ± 0.25	0.819
Redness (a*)	22.93 ± 0.65 ^b^	23.04 ± 0.70 ^b^	23.40 ± 0.45 ^a^	0.041
Yellowness (b*)	17.61 ± 0.29	17.48 ± 0.26	17.28 ± 0.24	0.472

^1^ Values are least squares means (LSM) ± standard error of the mean (SEM). ^a,b^ Means within a row with different superscript letters differ significantly (*p* < 0.05). ^2^ NS, natural suckling (positive control); MR, milk replacer (negative control); MR+BSR, milk replacer supplemented with *Boswellia serrata* resin (BSR).

## Data Availability

Data are contained within the article.

## Abbreviations

The following abbreviations are used in this manuscript:

ADG	Average Daily Gain
Alb	Albumin
ALT	Alanine Aminotransferase
AST	Aspartate Aminotransferase
BSR	*Boswellia serrata* Resin
BW	Body Weight
BUN	Blood Urea Nitrogen
Ca	Calcium
CF	Crude Fiber
CK	Creatine Kinase
CP	Crude Protein
Cr	Creatinine
DM	Dry Matter
DMI	Dry Matter Intake
EE	Ether Extract
FBW	Final Body Weight
FCR	Feed Conversion Ratio
GLOB	Globulin
Glu	Glucose
IBW	Initial Body Weight
LD	*Longissimus dorsi*
MR	Milk Replacer
NEFA	Non-Esterified Fatty Acids
NS	Natural Suckling
TBIL	Total Bilirubin
TP	Total Protein
TPA	Texture Profile Analysis
TWG	Total Weight Gain
WHC	Water-Holding Capacity
